# The Management of Recurrent Post-myelography Lumbar Pseudomeningocele With Epidural Blood Patch

**DOI:** 10.7759/cureus.35600

**Published:** 2023-02-28

**Authors:** Scott P Beckman, Carlie Proctor, Jamie B Toms

**Affiliations:** 1 Medicine, Louisiana State University (LSU) Health Shreveport, Shreveport, USA; 2 Neurosurgery, Louisiana State University (LSU) Health Shreveport, Shreveport, USA

**Keywords:** csf leak, laminectomy, myelography, epidural blood patch, pseudomeningocele

## Abstract

Pseudomeningoceles are a well-known potential postoperative complication of spinal and cranial surgeries that can occur after lumbar decompression and posterior fossa surgeries. They are often caused by incidental durotomies but may also occur as a result of dural puncture during diagnostic testing. This report describes a 59-year-old male that developed a recurrent pseudomeningocele after an L4 laminectomy for severe lumbar spinal stenosis that was ultimately treated with an epidural blood patch (EBP). His preoperative condition greatly improved, but he developed a pseudomeningocele that did not resolve after applying ice and light pressure. The patient subsequently underwent a wound exploration where no dural defect was identified. During this exploration, the dura was reinforced with dural onlays and sealant. Unfortunately, the patient developed another pseudomeningocele within a short interval. It was then suspected that the post-laminectomy site provided a space for the dural punctures from previous CT myelography to leak cerebrospinal fluid (CSF) into. The patient subsequently underwent ultrasound (US)-guided aspiration of the pseudomeningocele and EBP injections at the levels where his preoperative myelography was performed. The success of the EBP indicates that the previous CT myelography was the likely cause of the pseudomeningocele. Recurrent spinal pseudomeningoceles with no evidence of incidental durotomy may be caused by dural puncture from myelography. In such cases, EBP to the area that the previous myelography was performed can resolve the pseudomeningocele.

## Introduction

A pseudomeningocele is best defined as an extradural collection of cerebrospinal fluid (CSF) from a dural breach that is lined by a fibrous capsule [1]. Pseudomeningoceles are a potential complication of spinal surgery that may be asymptomatic or present with symptoms of positional headaches, neck stiffness, nausea, vomiting, dizziness, subcutaneous swelling, low back pain, or radiculopathy [2]. When a pseudomeningocele is clinically suspected, magnetic resonance imaging (MRI) is the preferred imaging modality for diagnosis [2]. Once a CSF intensity fluid collection is found on MRI and the patient has failed conservative therapy, surgical exploration of the area is warranted with dural repair if a defect is identified. However, if no dural defect is identified or the dural repair fails, epidural blood patch (EBP) can be performed [3]. Here, we report a case that illustrates myelography as the potential cause of a pseudomeningocele successfully treated with epidural blood patch after surgical exploration was unrevealing of a dural defect.

## Case presentation

**Figure 1:**
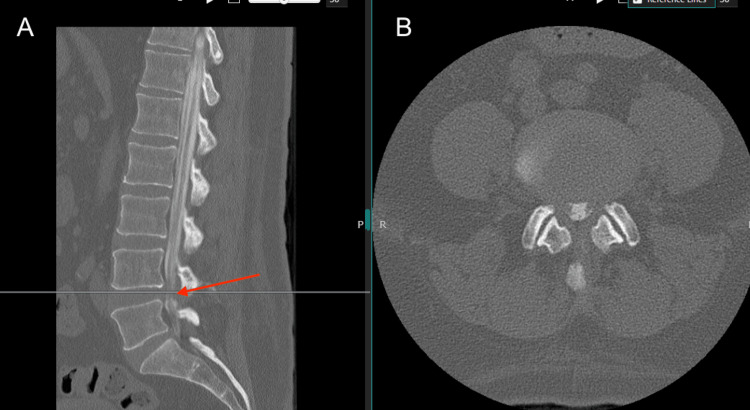
Preoperative CT Myelography Preoperative sagittal (A) and axial (B) myelography showing lumbar spinal stenosis at the level of L4/L5

**Figure 2:**
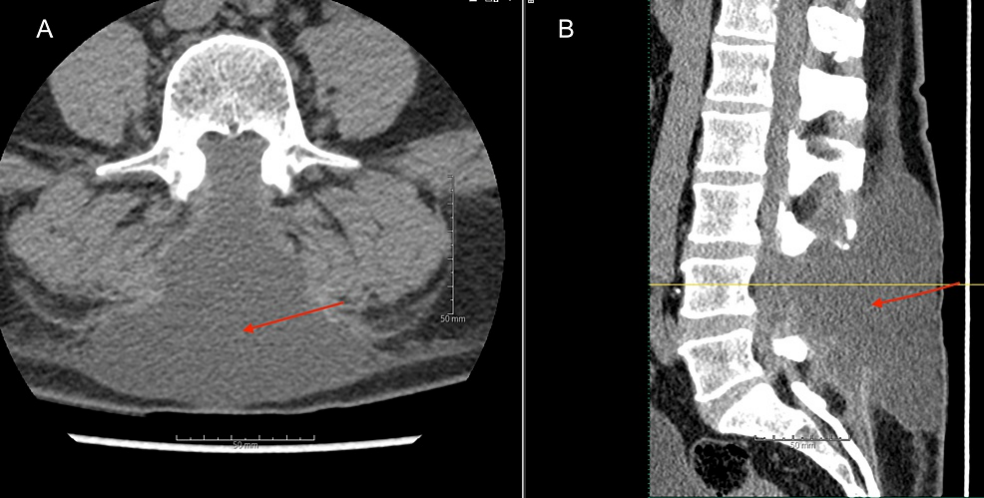
CT of the Lumbar Spine Without Contrast CT scan without contrast of the lumbar spine in the axial (A) and sagittal (B) planes showing a large, non-contrast-enhancing CSF intensity fluid collection within the soft tissue overlying the lumbar spine CSF: cerebrospinal fluid

A 59-year-old male with a non-MRI-compatible implanted cardiac defibrillator presenting with chronic neurogenic claudication and severe lumbar spinal stenosis diagnosed on CT myelography (Figure 1) whose symptoms failed to improve after conservative measures (physical therapy) underwent L4 laminectomy. The case was uncomplicated without dural breach, and the patient was discharged home postoperatively on day 0. Three weeks postoperatively, the patient was seen in the clinic with complaints of incisional swelling and non-positional headaches that began one week after the procedure. On examination, a large, firm, soft tissue edema was observed on the upper aspect of the incision. Otherwise, the incision was well-approximated and healing without suppuration or drainage. He was advised to apply ice and pressure over the site while reclining and to monitor for increased swelling. The patient then began having complaints of incisional pain and soreness, neck stiffness, nausea, fatigue, intermittent dizziness, and persistent headaches, now positional with significant improvement when lying flat. On examination, the swelling at the incision site was stable in size from the previous but soft and fluctuant. The history and examination findings raised concerns about a CSF leak. A lumbar CT scan with and without contrast was ordered to evaluate the wound. Imaging revealed a large, non-enhancing fluid collection concerning for pseudomeningocele (Figure 2).

The patient was admitted to the hospital and underwent wound exploration. Upon incision, a large amount of cerebrospinal fluid (CSF) was briskly expressed from the wound. The fascia and muscle were dehisced, and the dura was covered with scar tissue, but no durotomy was identified. The Valsalva maneuver was performed multiple times without the expression of CSF. Although no durotomy was identified, the dura was covered with dural onlay and dural sealant glue with watertight layered closure of the wound. Postoperatively, the patient remained flat for 24 hours and was later discharged home with follow-up. On postoperative day 4, he reported experiencing no positional headaches, neck stiffness, nausea, or dizziness.

Two weeks later, the patient had a return of the incisional fluid collection and persistent positional headaches. Subsequently, he developed clear incisional drainage and was admitted to the hospital for evaluation. Ultrasound (US)-guided aspiration was performed with a 9 × 14 × 9 cm fluid collection consistent with CSF. An epidural blood patch was performed at the sites of preoperative myelography, L1/L2 and L5/S1. The aspirated fluid was sent for culture yielding no growth. Following the completion of an epidural blood patch, the patient has not experienced a recurrence of his pseudomeningocele with the resolution of his symptoms.

## Discussion

Pseudomeningoceles are a well-known potential postoperative complication of lumbar laminectomy with an incidence estimated between 0.7% and 2%, although the true incidence is difficult to ascertain since many cases are asymptomatic [2,4,5]. Most pseudomeningoceles are iatrogenic and commonly arise after lumbar spine surgery as a result of incidental durotomy recognized during the procedure [5,6]. They may also present in a delayed fashion from several days to weeks after the procedure if dural tears go unnoticed intraoperatively [7]. Delayed onset may also occur after laminectomy if there is a bony spicule present along the margin of the laminectomy [3]. Pseudomeningoceles may also arise after a lumbar puncture or myelography needle puncture, which was the suspected etiology of our patient’s pseudomeningocele once it recurred after dural repair at the post-laminectomy site [1]. Clinical suspicion for postoperative pseudomeningoceles should be raised when patients present with symptoms of intracranial hypotension such as postural headaches and dizziness, as well as back pain or radiculopathy [8].

A vast majority of the literature on the management of postoperative pseudomeningoceles reports on those that are a result of intraoperative dural tears. The literature is lacking in reports on the successful management of pseudomeningoceles without evidence of a dural breach intraoperatively, i.e., a cryptogenic CSF leak, such as in the case of our patient. Commonly reported interventions for postoperative pseudomeningoceles include close observation for spontaneous resolution, bed rest for at least two days, lumbar subarachnoid drainage, abdominal binders, and surgical exploration and repair when conservative measures fail [9,10]. Solomon et al. described three cases of giant postoperative pseudomeningocele (over 8 cm in size) without an identifiable intraoperative dural tear that spontaneously resolved without surgical intervention [9]. Although spontaneous resolution has been reported to be a possibility for those with pseudomeningoceles, not all patients experiencing symptoms may be willing to exercise that option and would prefer a more active intervention.

An epidural blood patch (EBP) has been an effective treatment for post-dural puncture headaches and spontaneous intracranial hypotension with the first successful application reported in 1992 by Lauer and Haddox [10]. The mechanism of the therapeutic effect of an EBP is not currently known, but the theory is that it forms a gelatinous plug that seals the dural defect and prevents further CSF leakage [11]. Given that our patient’s pseudomeningocele was suspected to have been the result of dural punctures from previous CT myelography leaking CSF into his post-laminectomy site, EBP was the treatment of choice given the procedure’s known efficacy for successfully treating post-dural puncture headaches. Documented evidence is lacking for EBPs successfully treating postoperative lumbar pseudomeningoceles, with most of the evidence arising from small case reports, such as post-microdiscectomy patients with postoperative lumbar pseudomeningocele successfully treated with US-guided EBP published by Kavishwar et al. [10]. Additionally, Sandwell et al. reported the successful resolution of symptomatic postoperative lumbar pseudomeningocele with EBP injection in 16 of 19 patients for a success rate of 84%, providing a much-needed documentation of successful treatment with EBP in such patients [12]. This case further implicates the efficacy of EBP in the management of symptomatic pseudomeningocele, especially in the event of no identifiable dural defect.

## Conclusions

Recurrent post-spine surgery pseudomeningoceles with no identifiable CSF leak or evidence of incidental durotomy may be caused by dural puncture from previous myelography. In such cases, the successful resolution of pseudomeningocele may be achieved with the application of EBP to the area where the previous myelography or dural puncture was performed. This case provides evidence for the successful management of recurrent postoperative pseudomeningoceles when there is no clear evidence of a dural defect.
